# An analysis of disease-gene relationship from Medline abstracts by DigSee

**DOI:** 10.1038/srep40154

**Published:** 2017-01-05

**Authors:** Jeongkyun Kim, Jung-jae Kim, Hyunju Lee

**Affiliations:** 1 Gwangju institute of science and technology, School of Electrical Engineering and Computer Science, Gwangju, 61005, Korea; 2 1 Fusionopolis Way, #21–01 Connexis (South Tower), 138632, Singapore

## Abstract

Diseases are developed by abnormal behavior of genes in biological events such as gene regulation, mutation, phosphorylation, and epigenetics and post-translational modification. Many studies of text mining attempted to identify the relationship between gene and disease by mining the literature, but they did not consider the biological events in which genes show abnormal behaviour in response to diseases. In this study, we propose to identify disease-related genes that are involved in the development of disease through biological events from Medline abstracts. We identified associations between 13,054 genes and 4,494 disease types, which cover more disease-related genes than manually curated databases for all disease types (e.g., Online Mendelian Inheritance in Man) and also than those for specific diseases (e.g., Alzheimer’s disease and hypertension). We show that the text mining findings are reliable, as per the PubMed scale, in that the disease-disease relationships inferred from the literature-wide findings are similar to those inferred from manually curated databases in a well-known study. In addition, literature-wide distribution of biological events across disease types reveals different characteristics of disease types.

Identification of the genes involved in diseases is an important tool for revealing the molecular mechanisms of disease development and for the development of new drugs. Although existing web services, such as Online Mendelian Inheritance in Man (OMIM)^1^ and GeneCards^2^, identify genes related to diseases, these manually curated databases provide only a small percentage of the possible disease-gene relationships, once the rapid growth of scientific articles in the biomedical domain is taken into account. As a result, a number of text mining tools, such as PolySearch2^3^, DISEASES^4^, and DisGeNET^5^, have been developed to search for relationships between genes and diseases in the literature. PolySearch2^3^ uncovered latent associations among many types of biomedical entities by integrating a large collection of databases and several text mining algorithms, achieving an F measure of 88% for disease-gene association. DISEASES^4^ has extracted disease-gene associations from biomedical articles based on a text mining system with a comprehensive collection of dictionaries for human gene names, disease names, and their synonyms. It extracted 50% of manually curated associations with a 0.16% false-positive rate. DisGeNET^5^ collected more than 380,000 disease-gene associations by combining several curated databases with text-mined data. To assist the navigation of disease-gene associations from these various sources, it ranked the associations with prioritization scores. In addition to the above text mining tools providing the search engine functionality, several text mining methods to extract gene-disease relation from the literature have been developed. Various machine learning based methods, including a maximum entropy model^6^, conditional random field^7^, and support vector machine^8^, have been proposed. These methods were often combined with features from a dependency parser^8^ and several network properties from a disease-specific gene interaction network^9^, and the performances of these methods were tested using manually constructed corpora. However, because they were not applied to all PubMed abstracts, analysis of disease-related genes for all disease types were not provided. Although several tools support searches for which genes are related to which diseases, information about the biological events of genes that occur during disease development is rarely provided by these services. Biological events of genes, which might reflect abnormal behaviour due to diseases, include gene regulation, mutation, phosphorylation, and epigenetics and post-translational modification. To address this need, we adapt a text mining system, called DigSee, that searches for evidence sentences describing the “genes” involved in the development of “disease” through “biological events”. For example, the following evidence sentences show that up-regulation or mutation of a gene is related to disease: “Accordingly, up-regulation of GSK-3 may contribute to cytoskeletal pathology within neurites in AD”^10^ and “The GSTP1 A/G polymorphism was also associated with silicosis susceptibility”^11^.

While the previous version of DigSee^12^ is limited to cancer, we extend it to cover all disease types defined in Medical Subject Headings (MeSH) disease categories and to rank the disease-related genes based on the number of evidence sentences. As a result, we obtained disease-related genes for 4,494 MeSH disease types, including rare diseases such as silicosis and Costello Syndrome as well as common diseases such as hypertension. The text mining findings can be searched at http://gcancer.org/digsee.

We compared the identified disease-related genes with those from the OMIM^1^ and Genome Wide Association Studies (GWAS)^13^ databases, showing that the DigSee engine identifies more disease-related genes than those databases. We used Alzheimer’s disease and hypertension as case studies for comparing our results with disease-specific databases (e.g., AlzGene^14^ or the Text-mined Hypertension, Obesity and Diabetes (T-HOD) database^15^), demonstrating that DigSee not only augments these disease-specific databases with additional, relevant, disease-related genes, but also identifies more DrugBank^16^ drug target genes than these databases. When we investigated disease-disease relationships, based on the identified disease genes, our text mining results had statistical characteristics similar to the results of Menche *et al*.^17^ in their investigation of disease-disease relationships based on OMIM^1^ and GWAS studies^13^. This result provides further evidence for the accuracy of disease genes identified by our text mining approach.

## Results and Discussion

### Statistics of text mining results

**Figure 1:**
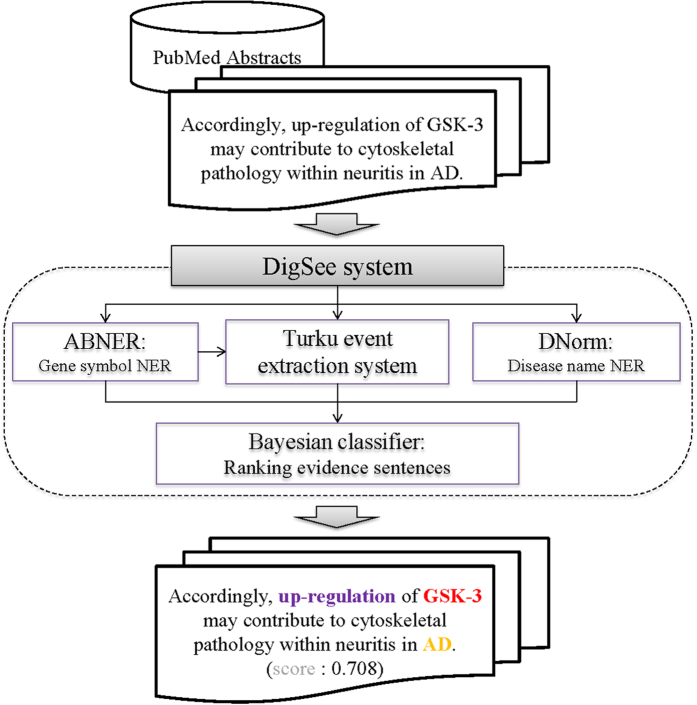
The Architecture of DigSee. Process of retrieving evidence sentences in DigSee: (i) collect sentences from PubMed abstracts; (ii) identify gene, event, and disease mentions from sentences; and (iii) calculate ranking of sentences based on the Bayesian model.

The extended DigSee search engine (Fig. 1) crawled Medline abstracts and collected sentences describing the relationship between genes and diseases through biological events. Gene and disease names were extracted from each sentence using named entity recognition (NER) tools. Biological events were identified using an event extraction system^18^. Sentences were then ranked by a Bayesian classifier (see the Methods section for details). DigSee retrieved over 13 million Medline abstracts from PubMed with disease names taken from the disease category descriptors in MeSH^19^. DigSee recognized and ranked a total of 7,303,284 evidence sentences from 8,513,732 abstracts and indexed them with 4,494 disease types, identifying disease associations for 13,054 genes.

**Figure 2:**
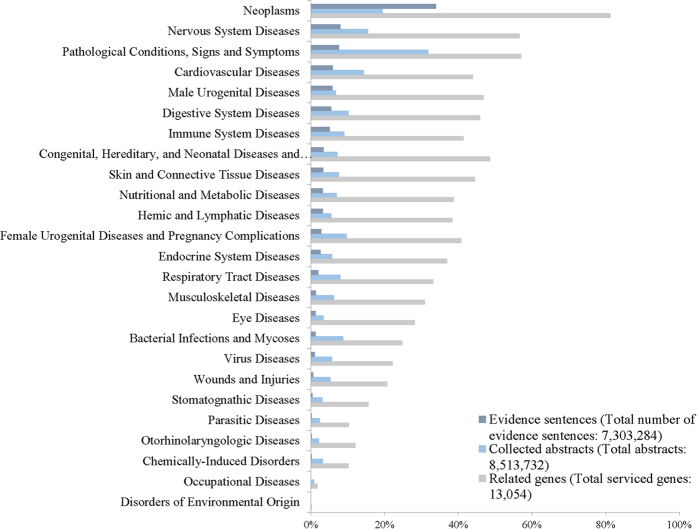
Distribution of the number of evidence sentences, the number of abstracts, and disease- related genes for general disease categories. A dark blue bar represents the percentage of evidence sentences out of the total number of evidence sentences for each disease category. For example, 34% of 7,303,284 sentences are related to neoplasms. A light blue bar and a gray bar represent percentages of abstracts and genes related to the given disease category, respectively. Note that abstracts and genes can belong to multiple disease categories.

Figure 2 and Supplementary Table 1 show the numbers of evidence sentences and retrieved genes for the most general 25 disease categories in MeSH. Note that DigSee only considers relationships between human genes and diseases. The animal disease category is not serviced in DigSee. Wounds and injuries are, however, included in DigSee because gene changes can arise by injury or as a part of the recovery process.

**Figure 3:**
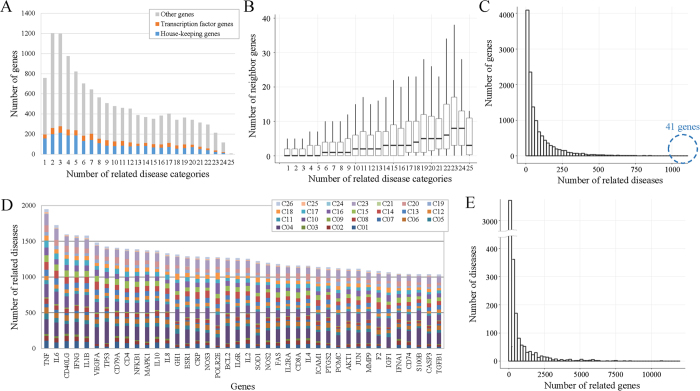
Statistics of disease-related genes identified by DigSee. (**A**) The number of genes in the 25 general Medical Subject Headings (MeSH) disease categories. (**B**) The numbers of neighboring genes in a protein interaction network for genes in the 25 general categories. (**C**) Genes are related with the different numbers of diseases. The *x*-axis represents the number of diseases involved with a gene and the *y*-axis represents the number of genes with that number of involved diseases. (**D**) For the top 41 genes related to more than 1000 diseases, the numbers of related diseases are shown. Bars are segmented by different colors according to the percentage of related general disease categories. C01–C26 represent the disease category identifiers used in MeSH. (**E**) Diseases are related to the different numbers of genes. The *x*-axis represents the number of genes related to a disease and the *y*-axis represents the number of diseases with that number of related genes.

We investigated which genes were involved in multiple disease types across the most general 25 categories in the MeSH ontology (Fig. 3A). With the exception of those genes involved in a single general category, the number of genes related to multiple categories tends to decrease as the number of categories increases. First, our analysis of housekeeping genes and transcription factors found that they are not related to a larger number of categories than other genes (Fig. 3A). Secondly, we found that those genes that interact with more genes in a protein interaction network are involved in more disease categories than those with fewer neighbors in the protein interaction network (Fig. 3B).

Figure 3C shows the number of genes involved in multiple, individual disease types rather than the 25 general disease categories. Forty-one genes are related to more than a thousand diseases, including TNF and interleukins. Figure 3D shows the distribution of their related disease types across the general categories. Figure 3E shows the number of diseases involving multiple genes. For example, for 320 diseases, which include carcinoma, leukemia, atherosclerosis, diabetes mellitus, breast cancer, and prostate neoplasms, more than 1000 genes were associated with the diseases. On the other hand, most diseases (around 78%) are associated with less than 200 genes, as reported in the literature.

### Comparison of identified disease-related genes with manually curated databases and other text mining tools

**Figure 4:**
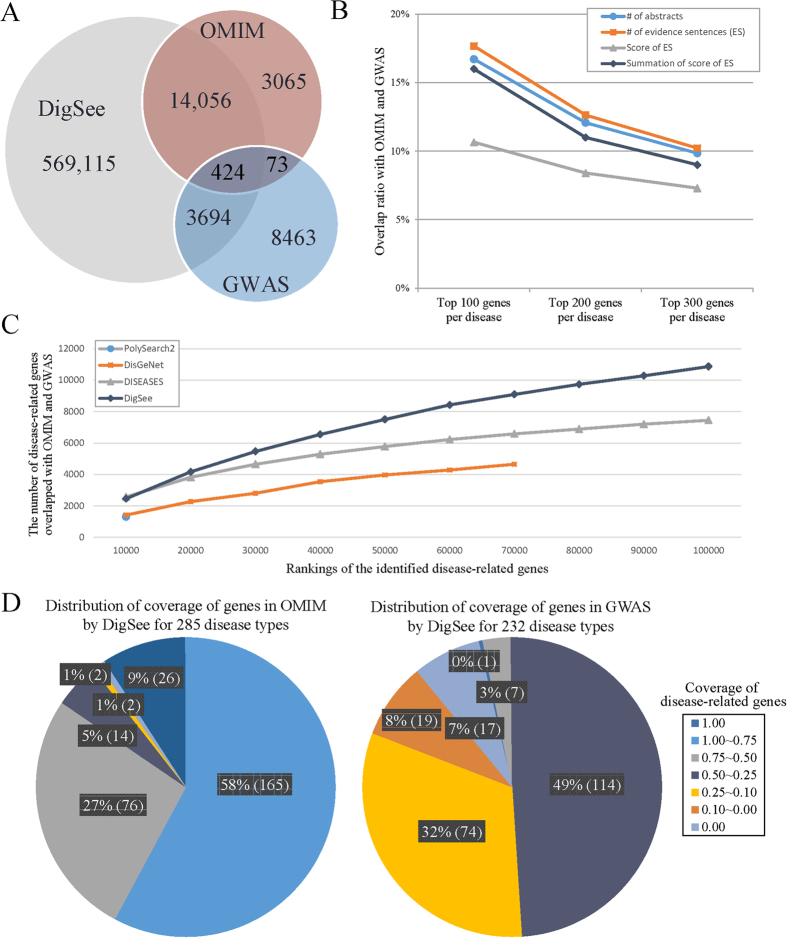
Common genes among Online Mendelian Inheritance in Man (OMIM), Genome Wide Association Studies (GWAS), and DigSee. (**A**) The number of common disease-gene relationships in OMIM, GWAS, and DigSee. (**B**) The overlap ratio of disease-related genes identified by DigSee among genes in OMIM and GWAS combined. For each disease, the top 100, 200, and 300 genes from the three ranking measures in DigSee are compared to the genes from OMIM or GWAS. (**C**) The number of overlapped genes between disease-related genes identified by DigSee, DISEASES, DisGeNet, and PolySearch2 and those from OMIM and GWAS. Disease-related genes were ranked by scores from each system. (**D**) For each disease, the ratio of genes in OMIM and GWAS identified by DigSee is calculated. The ratios are shown in the legend (e.g., 1.00–0.75 indicates that DigSee identified 75–100% of disease-related genes in OMIM or GWAS).

We compared genes from the OMIM^1^ and GWAS databases^13^ with disease-related genes identified by DigSee as follows: Menche *et al*.^17^ extracted genes related to 285 and 232 disease types from OMIM^1^ and GWAS studies^13^, respectively (all together 299 unique disease types), and we used them for the comparison. For the 299 disease types, DigSee identifies a total of 587,713 disease-gene associations. Of these, 14,480 and 4118 disease-gene associations were shared with OMIM and GWAS, respectively (Fig. 4A). We developed five measures to rank disease-related genes (see the Methods Section for details). We evaluated each of the five ranking measures by calculating the overlap ratio of its top K results (K = 100, 200, 300) with OMIM and GWAS, and found that the number of evidence sentences shows the best overlap ratio (18% from top 100 disease-related genes) (Fig. 4B; Supplementary Fig. 1; Supplementary Note). In fact, the performance of the two measures of the number of supporting abstracts and the summation of normalized scores is similar to that of the number of evidence sentences, while the other two measures of the highest score and the average score of evidence sentences do not perform well. These comparison results may indicate that the actual ranking is dependent on the frequency of evidence rather than the best or average evidence. We also applied thresholds to select evidence sentences based on the normalized scores of the evidence sentences: 0.2, 0.4, 0.6 and 0.8 (Supplementary Fig. 1C). Although the numbers of genes overlapped with OMIM and GWAS decrease with higher thresholds, the changes of overlap ratios were relatively small (from 18% to 12%) compared to the large number of reduced evidence sentences (from around 587,000 to around 98,000), showing the reliability of scores of evidence sentences.

For each of the 299 disease types, we also analyzed disease-related genes shared by OMIM, GWAS, and DigSee (Supplementary Table 2). We analyzed the statistical significance of overlaps between them with *p*-value < 0.05. DigSee had significant overlaps of genes for 99% of diseases (283/285) from OMIM and for 85% of diseases (198/232) from GWAS. Specifically, DigSee identified 75–100% of disease-related genes in OMIM for a majority of disease types (58%, 165/285), whereas it identified 50–75% of genes in GWAS for 49% (114/232) of diseases (Fig. 4D). As for the disease-related genes missed by DigSee, we found that genes or disease names were not always mentioned in abstracts (e.g., SCARA3 in dementia and MYO1E in nephritis), nor did the abstracts always describe the relationships between biological events and genes. For example, although RALGPS2 was known to be related to Alzheimer’s disease from a GWAS study, its biological events were not described in any PubMed abstract. Although DigSee recovered significant portions of genes covered in both the OMIM and GWAS databases, DigSee had more overlaps with OMIM. This may be because both OMIM and DigSee extracted disease-related genes from PubMed articles, whereas genes from GWAS studies were obtained from large-scale data sets, not all of whose genes were ever mentioned in research articles. Note that the difference in terms of overlaps is also consistent with the small number of overlaps of disease-gene relations between OMIM and GWAS.

We compared DigSee with three other disease-gene relationship search engines, PolySearch2, DISEASES, and DisGeNET, which identify disease-related genes by integrating disease databases or by employing text-mining approaches. For the 299 disease types from OMIM and GWAS, PolySearch2, DISEASES, and DisGeNet identify 9522, 174,193, and 74,746 disease-gene associations, respectively. Of these, 1304, 8748, and 4811 disease-related genes were overlapped with genes in OMIM and GWAS (Supplementary Fig. 2A), but the coverages of genes are smaller than that from DigSee. Because these tools provide confidence scores for the identified disease-related genes, we ranked disease-gene pairs and compared them to those in OMIM and GWAS. Figure 4C shows that DigSee has the highest overlap with genes in OMIM and GWAS compared to the other three tools across all rankings. In addition, for each disease type, we analyzed the statistical significance of overlaps between genes identified by these tools and those in OMIM and GWAS (Supplementary Fig. 2B and C). The numbers of disease types with p-value < 0.05 were the highest with DigSee, confirming better accuracies of DigSee.

### Case studies for Alzheimer’s disease and hypertension

**Figure 5:**
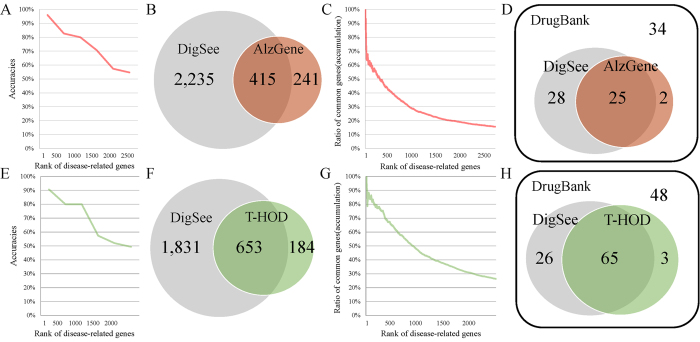
Comparison between existing databases for Alzheimer’s disease and hypertension and DigSee. (**A**) The accuracy of genes related to Alzheimer’s disease as identified by DigSee is shown by DigSee gene ranking. (**B**) The numbers of genes related to Alzheimer’s disease from AlzGene and DigSee and genes common to both. (**C**) Ratios of genes that are shared between AlzGene and DigSee, by DigSee gene ranking. (**D**) Comparison between 89 target genes of drugs for Alzheimer’s disease and genes from AlzGene and DigSee. (**E**) The accuracy of hypertension-related genes identified by DigSee is shown by DigSee gene ranking. (**F**) The numbers of hypertension-related genes from the Text-mined Hypertension, Obesity and Diabetes (T-HOD) database and DigSee, and genes common to both. (**G**) Ratios of common genes between T-HOD and DigSee, by DigSee gene ranking. (**H**) Comparison between 142 target genes of drugs for hypertension and genes from T-HOD and DigSee.

DigSee identifies 2650 genes related to Alzheimer’s disease (Supplementary Table 3). To confirm if these genes were related to Alzheimer’s disease, we randomly selected 75 genes for every 500 rankings (1–500, 500–1000, etc.) and manually validated whether they were related to Alzheimer’s disease according to their evidence sentences (Supplementary Table 4). Figure 5A shows that genes with higher rankings are more likely to be related to Alzheimer’s disease. Although more than 90% of the top 500 genes were correctly identified, only 55% of genes ranked between 2000 and 2500 were related to Alzheimer’s disease. We compared genes identified by DigSee with 656 genes in AlzGene^14^, an Alzheimer’s disease database, which collected comprehensive information about genes related to Alzheimer’s disease from genetic association studies. Figure 5B shows that 415 genes were shared between the two data sets.

We analyzed the most frequent 100 genes from DigSee in terms of the number of evidence abstracts, and found that 54 genes were present in AlzGene, and 42 of the remaining genes were confirmed to be related to Alzheimer’s disease based on evidence sentences, which reflects the accuracy of DigSee’s detection of genes related to Alzheimer’s disease. For details, refer to Supplementary Table 5. In addition, we confirmed that DigSee genes with higher rankings are more likely to be also found in AlzGene, as depicted in Fig. 5C. Note that the Y-axis in the figure indicates how many of the genes within a rank range are also found in AlzGene. Note that the accuracy of DigSee is above 55% even for the lower-ranked genes (Fig. 5A) and that AlzGene may miss some genes related to Alzheimer’s disease because the database does not collect genes as per the PubMed scale.

To see whether genes related to Alzheimer’s disease in DigSee are targets of drugs for Alzheimer’s disease, we collected 47 drugs for Alzheimer’s disease and 89 target genes of the drugs from DrugBank^16^, after excluding nutraceutical, illicit, and withdrawn genes/drugs in the database. When we compared the 89 targets with the genes in AlzGene and DigSee, AlzGene included only 30% (27/89) of the targets in DrugBank, whereas DigSee identified 60% (53/89) of the target genes (Fig. 5D and Supplementary Table 3).

We also investigated genes identified by DigSee for hypertension. We randomly selected 75 genes for every 500 rankings and checked whether they were related to hypertension (Supplementary Table 4). Figure 5E shows that genes with a higher ranking are more likely to be related to hypertension. We compared gene-disease relationships retrieved by DigSee with the text-mined T-HOD database^15^. T-HOD retrieved a list of genes relevant to hypertension, obesity, and diabetes using a text mining method. In T-HOD, the accuracy of the gene-disease relationships predicted for hypertension was 77% and the predicted results were manually validated up until 2011. T-HOD provides 837 hypertension-related genes, 653 of which overlap with DigSee (Fig. 5F). DigSee provided more genes (2484) than T-HOD. Genes with a higher ranking in DigSee are more likely to be also found in T-HOD (Fig. 5G). In addition, we collected 129 drugs associated with hypertension and 142 target genes of the drugs from DrugBank and compared the target genes with those from T-HOD and DigSee (Fig. 5H). T-HOD contained 47% (68/142) of targets from DrugBank, whereas DigSee supported 60% (91/142) (Supplementary Table 3).

From these two disease cases, we can see that DigSee’s coverage is higher than that of AlzGene and T-HOD. However, DigSee failed to identify a number of genes included in the two databases. We randomly selected and analyzed 100 of these genes for each of the two diseases in order to understand why they were not identified by DigSee (Supplementary Tables 6 and 7). We observed two main reasons. First, many disease-gene-biological event relationships are not explicitly expressed in abstracts, only in the full texts. For example, Giedraitis *et al*.^20^ support the relationship between APOA5 and Alzheimer’s disease in the full text, but not in the abstract. Secondly, A Biometrically Named Entity Recognizer (ABNER)^21^, which was used as a gene name recognition tool in DigSee, misses the mention of some genes. Furthermore, some recognized genes were incorrectly normalized by the gene name normalization tool, Moara^22^. Note that T-HOD has similar problems for gene name recognition and normalization because it was also constructed using text mining. About 60% of the 200 analyzed genes fall into the first and the second categories. Necessary future improvements to DigSee, therefore, include support for the full text of articles and improved gene name recognition and normalization.

### Disease-disease relationships based on disease-related genes

**Figure 6:**
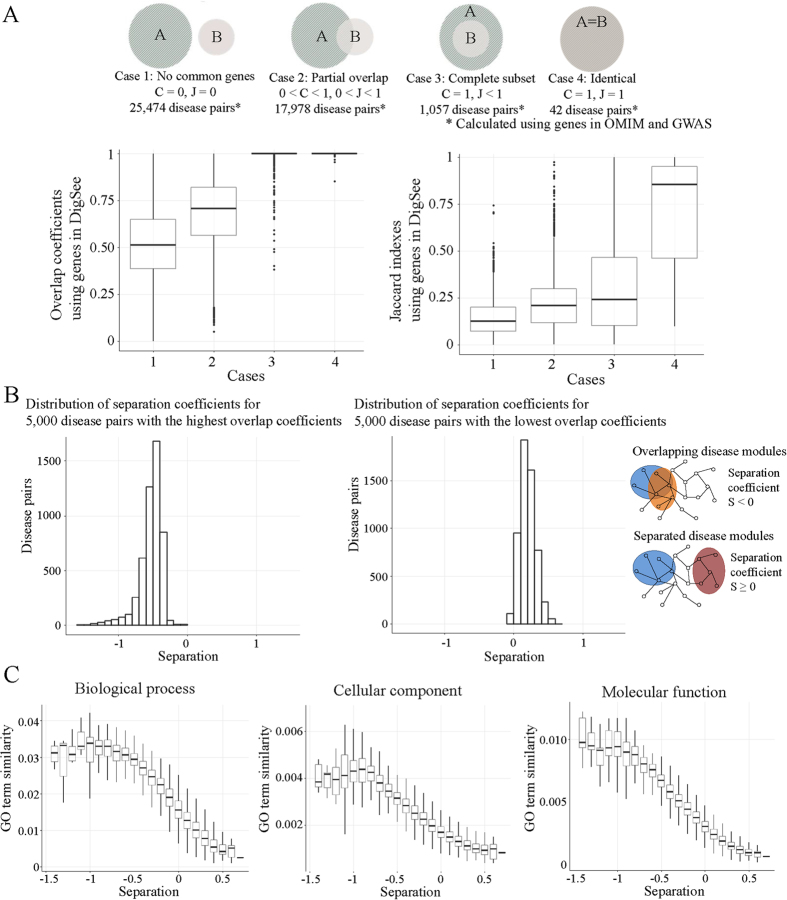
Relationships between diseases. (**A**) Disease pairs were classified into four groups using genes in OMIM and GWAS. For these disease pairs, the overlap coefficients and Jaccard indices were calculated using genes in DigSee. (**B**) Distributions of separation coefficients of disease pairs in the protein interaction network are different for two groups of disease pairs: 5000 disease pairs having the highest overlap coefficients, and 5000 disease pairs having the lowest overlap coefficients. Negative values of separation coefficients S indicate that genes are close in the protein interaction network, whereas positive values indicate that genes are separate in the network. (**C**) Disease pairs that are closer in the protein interaction network (with smaller separation coefficients) are more likely to have high gene ontology (GO) term similarities for biological process, cellular component, and molecular function.

Menche *et al*.^17^ investigated disease-disease relationships based on disease-related genes and a protein interaction network. They found that some diseases perturb similar biological pathways by containing the same genes or genes that are closely connected in a protein interaction network, resulting in similar clinical and pathological conditions. They obtained disease-related genes from the OMIM^1^ and GWAS databases^13^ and then calculated the similarities between diseases by computing the overlap coefficient 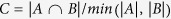 and the Jaccard index 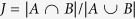, where |*A*| and |*B*| represent the numbers of disease-related genes in diseases *A* and *B*, respectively. The higher values of *C* and *J* represent more closely related diseases. The disease pairs used by Menche *et al*.^17^ were grouped into four cases: complete separation, partial overlap, complete subset, and identical, as illustrated at the top of Fig. 6A. In addition, the separation coefficient representing network-based separation of a disease pair is calculated as 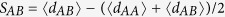, where 〈*d*_*AB*_〉 represents the shortest distance between gene pairs from diseases *A* and *B* in the protein interaction network. If a gene is commonly related to diseases *A* and *B*, 〈*d*_*AB*_〉 = 0. Menche *et al*.^17^ showed that disease pairs with larger *C* and *J* values have smaller separation coefficients of *S*_*AB*_, confirming that the incomplete protein interaction network can reveal disease-disease relationships. Furthermore, it was shown that disease pairs predicted as being closely related to each other based on the *C, J*, and *S*_*AB*_ scores were functionally similar.

Inspired by their work, we investigated whether the disease-related genes identified by DigSee present similar disease-disease relationships as those in Menche *et al*.^17^. Because DigSee covers more disease-related genes than OMIM and GWAS, as described in the previous section, the numbers of disease pairs that were completely separated (13) or identical (5) in DigSee were small. We calculated the overlap coefficient *C* and the Jaccard index *J* with the disease pairs from Menche *et al*.^17^ using the disease-related genes from DigSee. We noted that the overlaps between disease pairs were higher for the complete subset group and the identical group than those for partial overlap and complete separation (Fig. 6A). When we calculated the separation coefficients using genes identified by DigSee, we found that disease pairs were more separated in the protein interaction network when their overlap coefficients *C* were smaller (Fig. 6B), showing that disease-related genes in DigSee have similar properties to genes in the OMIM and GWAS databases.

We also tested whether the disease-related genes identified by DigSee could be used to reveal functional relationships between diseases. We calculated the functional similarity of disease-related genes by gene ontology (GO) annotations, in the same manner as Menche *et al*.^17^. The similarity between genes *a* and *b, S*_*GO*_(*a, b*) is calculated based on the most specific GO term *i* among those shared by the two genes. *S*_*GO*_(*a, b*) ≡ 2/min(*n*_*i*_), where *n*_*i*_ is the total number of genes annotated for the GO term *i*. Then, the similarity of two diseases *A* and *B* is determined by the average of GO similarity of all gene pairs in diseases *A* and *B*. Figure 6C shows inverse relationships between GO term similarities and separation coefficients, indicating that given two diseases, the more their related genes are separated in the protein interaction network, the less their related genes are likely to have high GO term similarities. When we calculated the separation coefficients and GO term similarities for Fig. 6B and C, we used the top 100 genes for each disease ranked by the number of evidence sentences. Similarly, when we calculated the separation coefficients and GO term similarities using the top 200 and 300 genes and other measures (the numbers of abstracts and the scores of evidence sentences), we obtained similar relationships between diseases, indicating that highly ranked disease-related genes reliably explain the relationship between diseases (Supplementary Fig. 3 and Supplementary Tables 8, 9, 10 and 11).

In summary, for uncovering disease-disease relationships and functional relationships between diseases, the disease-related genes text-mined by DigSee present similar results as those identified from OMIM and GWAS, which supports reliabilities of the identified disease-related genes.

### Comparing biological events with regard to their associated disease types

**Figure 7:**
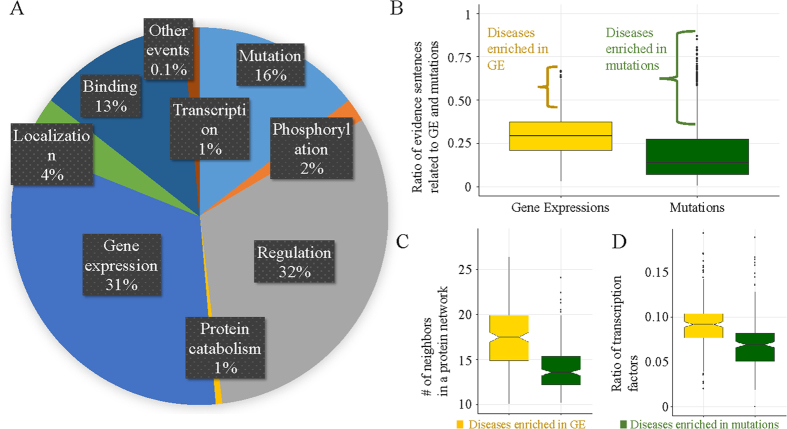
Analysis of biological events for diseases. (**A**) Statistics of biological events for diseases. (**B**) Distribution of ratio of evidence sentences related to gene expressions and mutations. (**C**) Distribution of the numbers of neighboring genes in a protein network. (**D**) Distribution of ratio of transcription factor genes.

Figure 7A shows the frequency of each biological event type in DigSee. We investigated how these biological events are distributed across disease types (Supplementary Table 12). As shown in Fig. 7B, some disease types have more events of either gene expression or mutation and thus fewer events of the other type. For example, disease types such as cleft lip, deafness, and hearing loss were more frequently associated with mutations than gene expression events. More specifically, for 197 disease types, >36% (=*μ* + 1*σ*) of evidence sentences were related to mutations, referred to as mutation-enriched diseases. For 198 disease types, >41% (=*μ* + 1*σ*) of evidence sentences were related to expression, referred to as gene expression-enriched diseases (Fig. 7B).

This difference leads us to the hypothesis that it might be difficult in practice to study expression level changes and regulations in some diseases, but relatively easier to check mutations based on family studies. As a consequence, these diseases may have been associated with relatively smaller numbers of genes and their molecular mechanisms may have been less clearly revealed than the other diseases. To support the hypothesis, we examined the genes related to mutation-enriched diseases. Figure 7C shows that genes related to mutation-enriched diseases have significantly fewer neighboring genes in the protein network than those related to the gene expression-enriched diseases (*p*-value = 5.10E-26). Similarly, it was observed that the ratio of transcription factors among genes related to mutation-enriched diseases was less than that of genes related to the gene expression-enriched diseases, with a *p*-value of 6.23E-13 (Fig. 7D).

### Evaluation of rankings of evidence sentences for extended biological events and disease types

Previously, we developed a Bayesian model for ranking evidence sentences for seven biological events using cancer-related abstracts^12^. In the current study, we extended events to include those of the epigenetics and post-translational modification (EPI) task from the BioNLP shared tasks and mutations. The Bayesian model was also tested for other diseases (Supplementary Tables 13, 14, 15 and 16). When we randomly selected 73 evidence sentences for the EPI task events and tested their accuracy (Supplementary Table 13), we achieved an area under the curve (AUC) value of 70.2% and an F score of 69.5% (Supplementary Table 17). The performance of the Bayesian classifier was also tested for the mutation event using 197 evidence sentences (Supplementary Table 14). Evidence sentences for mutation events were classified as positive sentences when the gene with the retrieved mutation was involved in a disease and as negative when the gene was not involved. The accuracy of mutation events was estimated at an AUC value of 62.3% and an F score of 81.6% (Supplementary Table 17).

In addition, we tested whether the Bayesian model developed for cancer in our previous study^12^ could be applied to other diseases. We constructed training and test evidence sentences for Alzheimer’s disease (525 sentences in Supplementary Table 15). For the disease, we compared two Bayesian models–a model trained with the cancer dataset and another model trained with the training dataset of Alzheimer’s disease–where both models were evaluated against the test dataset of Alzheimer’s disease. When evaluated against the Alzheimer’s disease data, the model trained with the cancer dataset showed an AUC value of 76.8% and an F score of 72.6% (Supplementary Table 18), which was similar to that of the model trained with the Alzheimer’s disease training set (an F score of 71.0%; 5-fold cross-validation). In Supplementary Table 18, the accuracy of some biological events was not measured due to lack of negative or positive evidence sentences.

Similarly, we tested the model against 268 sentences for hypertension (Supplementary Table 16). When evaluated against the hypertension data, the model trained with the cancer data showed an AUC value of 69.2% and an F score of 65.4% (Supplementary Table 18), which was similar to the performance of the model trained with the hypertension training data (an F score of 67.1%; 5-fold cross-validation).

## Methods

The all-disease version of DigSee is based on the previous cancer version^12^, which has the following steps: collection of relevant Medline abstracts, event extraction using text mining tools, and disease-related gene ranking. Our works for the extension include not only the collection of Medline abstracts related to the MeSH disease categories as explained in the Results and discussion section, but also normalizing disease names from the Medline abstracts, including more event types in order to increase the coverage of events, developing disease-independent methods for generating the machine learning features of evidence sentence ranking, and ranking disease-related genes.

### Disease name normalization

We use the disease names of the MeSH^19^ disease categories for the collection of Medline abstracts. If a disease has multiple names, searching with a name of the disease may miss the occurrences of the other names of the disease. To resolve this issue, we adopt DNorm^23^, which normalizes diverse disease names into standard terms.

### Increasing the coverage of events and evaluating text mining tools for disease-gene relationship identification

DigSee utilizes the Turku event extraction system (TEES)^18^ to locate biological events in Medline abstracts. While the previous cancer version targets seven core biological event types of BioNLP shared tasks (BioNLP-ST) in 2009 (i.e., gene expression, regulation, protein catabolism, phosphorylation, localization, binding, and transcription), the all-disease version additionally targets seven types of the EPI task from BioNLP-ST 2011 (i.e., hydroxylation, ubiquitination, DNA methylation, glycosylation, acetylation, methylation, catalysis, and their reverse reactions with the exception of catalysis) in order to increase the coverage of events. DigSee combines a reaction with its reverse reaction (e.g., phosphorylation-dephosphorylation) into a single event type, since reaction directionality does not affect disease-related gene ranking. In addition, it utilizes tmVar^24^ to locate mutation events. In total, DigSee all-disease version locates 15 types of biological events. The Turku event extraction system was reported to achieve a precision of 53.98%, a recall of 52.69%, and an F measure of 53.33% at the EPI task in BioNLP-ST 2011^25^. tmVar was reported to achieve an F measure of 91.39% (precision of 91.38% and recall of 91.40%). To confirm if the performance is consistent with the Medline abstracts relevant to our study, we re-evaluated TEES and tmVar and the other text mining tools, including ABNER^21^ for gene name identification and Moara^22^ for gene name normalization, against sample Medline abstracts of our experiments and found similar results (see Supplementary Note for details).

### Disease-independent methods for generating features of evidence sentence ranking

The previous cancer version of DigSee introduced a Bayesian model for identifying evidence sentences, based on linguistically motivated features, and used the confidence scores of evidence sentences estimated by the model for ranking disease-related genes. Among the features, two features of “cancer keywords count” and “hallmark keywords count” were based on hand-crafted cancer-related terms and thus disease-dependent. To apply the two features for all diseases, we develop a novel disease-independent method of collecting disease-related terms by using Word2Vec^26^. Word2Vec computes continuous vector representations of words based on neural networks, where the word vectors can be used for certain inference, for example, with a known pair of (‘king’, ‘queen’) and a given word ‘man’, a simple vector operation on the word vectors (i.e. vector(‘king’) − vector(‘queen’) + vector(‘man’)) can deduce ‘woman’. We adopt Word2Vec to learn vector representations of words in Medline abstracts and to predict disease-related terms as follows: We have pairs of (cancer, cancer-related term) from the previous cancer version (e.g. (‘cancer’, ‘proliferation’)). For a given disease (e.g. ‘hypertension’), we compute vector(cancer) − vector(cancer-related term) + vector(given disease) to predict terms related to the given disease (see Supplementary Note for details of the Bayesian model).

We also tested whether the feature weights of the Bayesian model trained with the cancer data^12^ could be applied to other diseases. We measured the accuracy of the extracted evidence sentences for two diseases (Alzheimer’s disease and hypertension) and found that it is similar to the accuracy of cancer-related evidence sentences. This supports that the feature weights learned from the cancer data can be used for other diseases.

### Improving disease-related gene ranking

We compared five measures for ranking genes for each disease, including 1) the number of abstracts supporting disease-gene relations, 2) the number of evidence sentences, 3) the highest score of evidence sentences, and 4) the summation and 5) the average of normalized scores of evidence sentences. The first measure counts the number of abstracts containing the evidence sentences with disease-gene-biological events for each gene. The second measure counts the number of evidence sentences for each gene. Note that when calculating the first two measures, the scores of evidence sentences were not considered. The third measure selects the highest among the scores of the evidence sentences of a gene. For the fourth and fifth measures, genes are ranked by the summation and the average of normalized scores of the evidence sentences of the genes, respectively. We normalized scores using a sigmoid function.

### The DigSee web interface

**Figure 8:**
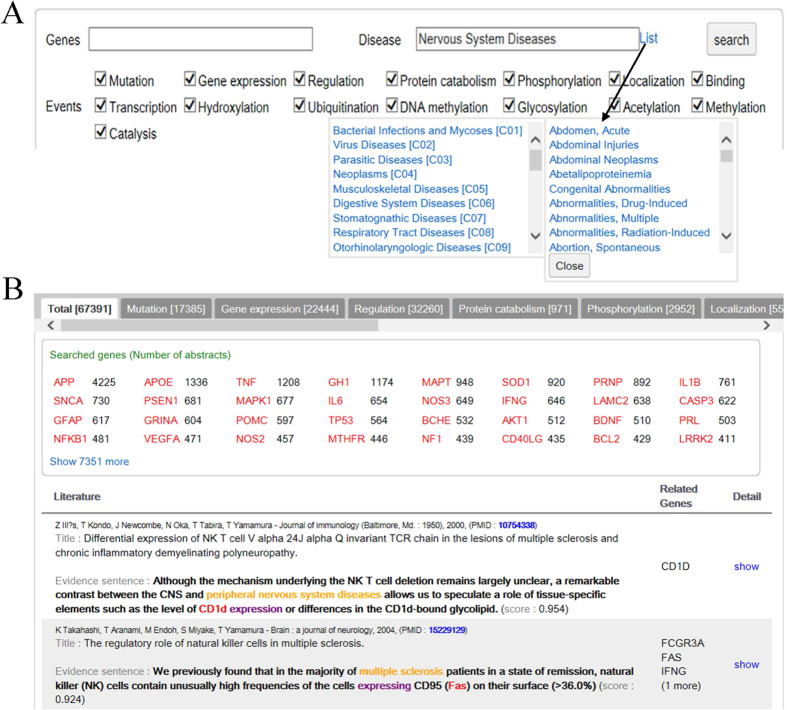
The DigSee interface overview. (**A**) A web interface of DigSee. Users input gene symbols (optional) and a disease name, and select biological events. (**B**) Part of search results for nervous system disease.

DigSee’s query consists of the following elements: (i) disease of interest (a list of disease terms from the MeSH^19^ disease categories is provided for search and selection); (ii) a list of gene(s) (if no gene is given in the query, all genes are considered as candidate results); and (iii) biological event type(s) from 15 possible event types. These are illustrated in Fig. 8A. Search results consist of a list of disease-related genes and Medline abstracts with evidence sentences (Fig. 8B).

## Additional Information

**How to cite this article**: Kim, J. *et al*. An analysis of disease-gene relationship from Medline abstracts by DigSee. *Sci. Rep.* **7**, 40154; doi: 10.1038/srep40154 (2017).

**Publisher's note:** Springer Nature remains neutral with regard to jurisdictional claims in published maps and institutional affiliations.

## Supplementary Material

Supporting Information

Supplementary Table 1

Supplementary Table 2

Supplementary Table 3

Supplementary Table 4

Supplementary Table 5

Supplementary Table 6

Supplementary Table 7

Supplementary Table 8

Supplementary Table 9

Supplementary Table 10

Supplementary Table 11

Supplementary Table 12

Supplementary Table 13–16

Supplementary Table 17

Supplementary Table 18
