# Corrigendum: An analysis of disease-gene relationship from Medline abstracts by DigSee

**DOI:** 10.1038/srep46437

**Published:** 2017-04-11

**Authors:** Jeongkyun Kim, Jung-jae Kim, Hyunju Lee

Scientific Reports 7: Article number: 4015410.1038/srep40154; published online: 01 05 2017; updated: 04 11 2017

In this Article, Jung-jae Kim is incorrectly listed as being affiliated with ‘1 Fusionopolis Way, #21–01 Connexis (South Tower), 138632, Singapore’.

The correct affiliation is listed below:

Institute for Infocomm Research, Data Analytics Department, 138632, Singapore.

