# Supplementary material for: An analysis of disease-gene relationship from Medline abstracts by DigSee
Source: Sci Rep. 2017 Jan 5;7:40154. doi: 10.1038/srep40154 (PMC5215527; doi:10.1038/srep40154)

An analysis of disease-gene relationship from Medline abstracts by DigSee

Jeongkyun Kim[1], Jung-jae Kim[2], and Hyunju Lee[1,*]

[1]Gwangju institute of science and technology, School of Electrical Engineering and Computer Science, Gwangju, 61005, Korea
[2]1 Fusionopolis Way, #21–01 Connexis (South Tower), Singapore 138632
* hyunjulee@gist.ac.kr

Supporting Information

Supplementary Note. Re-evaluation of text mining tools and detailed ranking method of DigSee

Supplementary Figure 1. Overlap ratio of disease-related genes between OMIM, GWAS, and DigSee with top ranked genes.

Supplementary Figure 2. Comparison results of DigSee and other text mining tools with OMIM and GWAS.

Supplementary Figure 3. Separation coefficients and gene ontology (GO) term similarities between genes using top-ranked genes.

Supplementary Table 1. Statistics of evidence sentences, disease-related genes, and abstracts for general disease categories.

Supplementary Table 2. Commonly identified disease-related genes from Online Mendelian Inheritance in Man (OMIM), Genome-Wise Association Studies (GWAS), and DigSee.

Supplementary Table 3. Lists of disease-related genes from AlzGene, the Text-mined Hypertension, Obesity and Diabetes (T-HOD) database, DrugBank, and DigSee.

Supplementary Table 4. Manual validation of genes related to Alzheimer’s disease and hypertension in DigSee.

Supplementary Table 5. Top 100 genes related to Alzheimer’s disease in DigSee and validation of these genes.

Supplementary Table 6. Analysis of genes related to Alzheimer’s disease that were not identified by DigSee but found in AlzGene.

Supplementary Table 7. Analysis of genes related to hypertension that were not identified by DigSee but found in T-HOD.

Supplementary Table 8. Overlap coefficients, jaccard indices, and separation coefficients between disease pairs using all disease-related genes in DigSee.

Supplementary Tables 9 - 11. Overlap coefficients, jaccard indices, separation coefficients, and GO similarities between disease pairs using the top 100, 200, or 300 disease-related genes in DigSee.

Supplementary Table 12. Ratio of mutations and gene expressions among biological events for each disease type.

Supplementary Tables 13-16. Gold standard evidence sentences for Alzheimer's disease, hypertension and extended biological events.

Supplementary Table 17. Performances of the Bayesian model for ranking evidence sentences for EPI biological events and mutation.

Supplementary Table 18. Performances of the Bayesian model for ranking evidence sentences for Alzheimer's disease and hypertension.

Supplementary Note

Re-evaluation of text mining tools used in DigSee

DigSee used several state-of-the-art text mining tools to identify disease-gene relations. To confirm if the performance is consistent with the Medline abstracts relevant to our study, we re-evaluated TEES and the other text mining tools against 100 sample Medline abstracts of our experiments and found similar results as follows:

A. Turku event extraction system

DigSee utilizes the Turku event extraction system (TEES)[1] to locate biological events in Medline abstracts. It was reported to achieve a precision of 53.98%, a recall of 52.69%, and an F measure of 53.33% at the EPI task in BioNLP-ST 2011.[2] We tested TEES with randomly selected 100 Medline abstracts. In the sample abstracts, TEES extracted total 1177 event relations, 55.14% of which were identified correct (679 relations) in both named-entity recognition (NER) and event relation. Among the incorrect relations, 83 relations were attributed to the errors of ABNER.

B. ABNER

ABNER,[3] the NER tool for identifying gene and protein mentions, shows a precision of 77.93% in sample Medline abstracts. The accuracy of ABNER was previously known to achieve an F- measure of 69.9% in the previous study.[3] We also compared BANNER[4] and Gimli[5] with ABNER in the DigSee pipeline. In the validation results, from the 100 abstracts, ABNER and BANNER extracted 1,278 and 1,265 gene names with similar accuracies (ABNER: a precision of 77.93% and BANNER: a precision of 76.44%). Although the precision of Gimli was higher as 82.57% than the other two tools, it identifies the smaller number of genes names (1,033). Based on these empirical results, we used ABNER as the NER tool.

C. DNorm

We use DNorm[6] for extracting disease mentions and normalizing diverse disease names into standard terms. In the results of sample Medline abstracts that randomly selected 100 abstracts, DNorm recognized 778 disease mentions, 76.34% of which were identified correct in both NER and normalization. DNorm was known to achieve an F-measure of 80.9% against a test set of the NCBI disease corpus in the previous study.[6]

D. tmVar

For locating mutation event, DigSee utilizes tmVar.[7] tmVar was reported to achieve an F-measure of 91.39% (a precision of 91.38% and a recall of 91.40%). tmVar achieved a precision of 99.58% from randomly selected 100 sample Medline abstracts. In the sample abstracts, tmVar recognized total 726 mutation mentions.

E. Moara

Moara[8] is a flexible and trainable text mining system for gene/protein tagger and normalization. The system has been trained for several model organisms and corpora, moreover it can be expanded to support new organisms and documents. DigSee utilizes the Moara to normalize recognized gene mentions with human model, but not used to recognize gene and protein mentions. Moara achieved a precision of 55.00%, a recall of 83.31%, and an F-measure of 66.26% in the previous study.[8] In the randomly selected 100 Medline abstracts, Moara achieved a precision of 75.10% in the normalization step.

A ranking method for evidence sentences

To distinguish positive sentences supporting the triplet relationship of gene, disease, and biological event from negative sentences that do not describe the relationship, we previously developed a Bayesian model based on ten linguistically motivated features were constructed using the feature selection sentences[9] such as event and edge scores, gene-event distance, event-regulation distance, and event-disease distance, event depth, cancer keywords count, hallmark keywords count, negative score, and agent. These features were obtained from ABNER[3] and Turku event extraction system[1], dependency parse trees generated by Stanford parser, hand-crafted disease-related

terms, and terms related to negative sentences.

Developed Bayesian classifier with the features was modeled to identify positive evidence sentences from negative sentences. By assigning the same prior to positive and negative evidence sentences, we calculated a likelihood ratio of features,

$$\mathrm{L(features)} = \frac{p(\mathrm{features}|\mathrm{positive})}{p(\mathrm{features}|\mathrm{negative})}$$

Basically, naïve Bayesian classifier assumes conditional independence among features. However, we empirically chose two types of dependencies, a dependency between cancer keyword and event-cancer distance and a dependency between agent and hallmark keywords count, after analyzing the feature selection data set. Therefore, the likelihood ratio can be rewritten as follows:

$$L(\mathrm{features}) = \prod_{\mathrm{features}_i} \frac{p(\mathrm{features}_i|\mathrm{positive})}{p(\mathrm{features}_i|\mathrm{negative})} \cdot \frac{p(\text{cancer keywords count}|\text{positive, event-cancer distance})}{p(\text{cancer keywords count}|\text{negative, event-cancer distance})} \cdot \frac{p(\text{agent}|\text{positive, hallmark keywords count})}{p(\text{agent}|\text{negative, hallmark keywords count})}$$

Among the features, two features of “cancer keywords count” and “hallmark keywords count” were based on hand-crafted disease-related terms that means disease-dependent features. To adopt two features for all diseases, we develop a method of collecting disease-related terms using Word2Vec.[10] Word2Vec computes continuous vector representations of words based on neural networks, where the word vectors can be used for certain inference. At first, we computed the vectors of words using all sentences in disease-related Medline abstracts. The ten most similar words of each disease name in the vector space were selected as disease keywords. In addition, we identified hallmarks of a given disease (e.g., hypertension) by contrasting them with known pairs of cancers and their hallmarks (e.g., proliferation). In particular, we used vector operations such as $\mathrm{vector(“cancer”)} - \mathrm{vector(“proliferation”)} + \mathrm{vector(“hypertension")}$ to predict terms related to the given disease. For example, term “blood pressure” was found to be the top hallmark of hypertension using example vector operation.

Additional methods for ranking disease-related gene ranking

In addition to the five measures introduced in “Improving disease-related gene ranking” in the Method section, we augmented the fourth and fifth measures with biological events in order to see whether particular biological events might affect gene ranking and generated four additional ranking. First, for each disease, the ratios of biological events were calculated, and the summation and the average of normalized scores were multiplied by the event ratio (6th and 7th rankings). Second, for each gene, event ratios were calculated and the summation and the average of normalized scores were multiplied by the event ratio of genes (8th and 9th rankings) (Supplementary Figure 1).

References

1. Bj¨orne, J. & Salakoski, T. Generalizing biomedical event extraction. In *Proceedings of the BioNLP Shared Task 2011 Workshop*, 183–191 (2011).

2. Ohta, T., Pyysalo, S. & Tsujii, J. Overview of the epigenetics and post-translational modifications (epi) task of bionlp shared task 2011. In *Proceedings of the BioNLP Shared Task 2011 Workshop*, 16–25 (2011).

3. Settles, B. Abner: an open source tool for automatically tagging genes, proteins and other entity names in text. *Bioinformatics* **21**, 3191–3192 (2005).

4. Leaman, R., Gonzalez, G. et al. Banner: an executable survey of advances in biomedical named entity recognition. In *Pacific symposium on biocomputing*, vol. 13, 652–663 (2008).

5. Campos, D., Matos, S. & Oliveira, J. L. Gimli: open source and high-performance biomedical name recognition.

BMC Bioinformatics **14**, 54 (2013).

6. Leaman, R., Islamaj Do˘gan, R. & Lu, Z. Dnorm: disease name normalization with pairwise learning to rank. *Bioinformatics* **29**, 2909–2917 (2013).

7. Wei, C.-H., Harris, B. R., Kao, H.-Y. & Lu, Z. tmvar: a text mining approach for extracting sequence variants in biomedical literature. *Bioinformatics* **29**, 1433–1439 (2013).

8. Neves, M. L., Carazo, J.-M. & Pascual-Montano, A. Moara: a java library for extracting and normalizing gene and protein mentions. *BMC Bioinformatics* **11**, 157 (2010).

9. Kim, J. et al. Digsee: disease gene search engine with evidence sentences (version cancer). *Nucleic Acids Research* **41**, W510–W517 (2013).

10. Mikolov, T., Chen, K., Corrado, G. & Dean, J. Efficient estimation of word representations in vector space. *arXiv:1301.3781* (2013).

Supplementary Figure 1

(A)

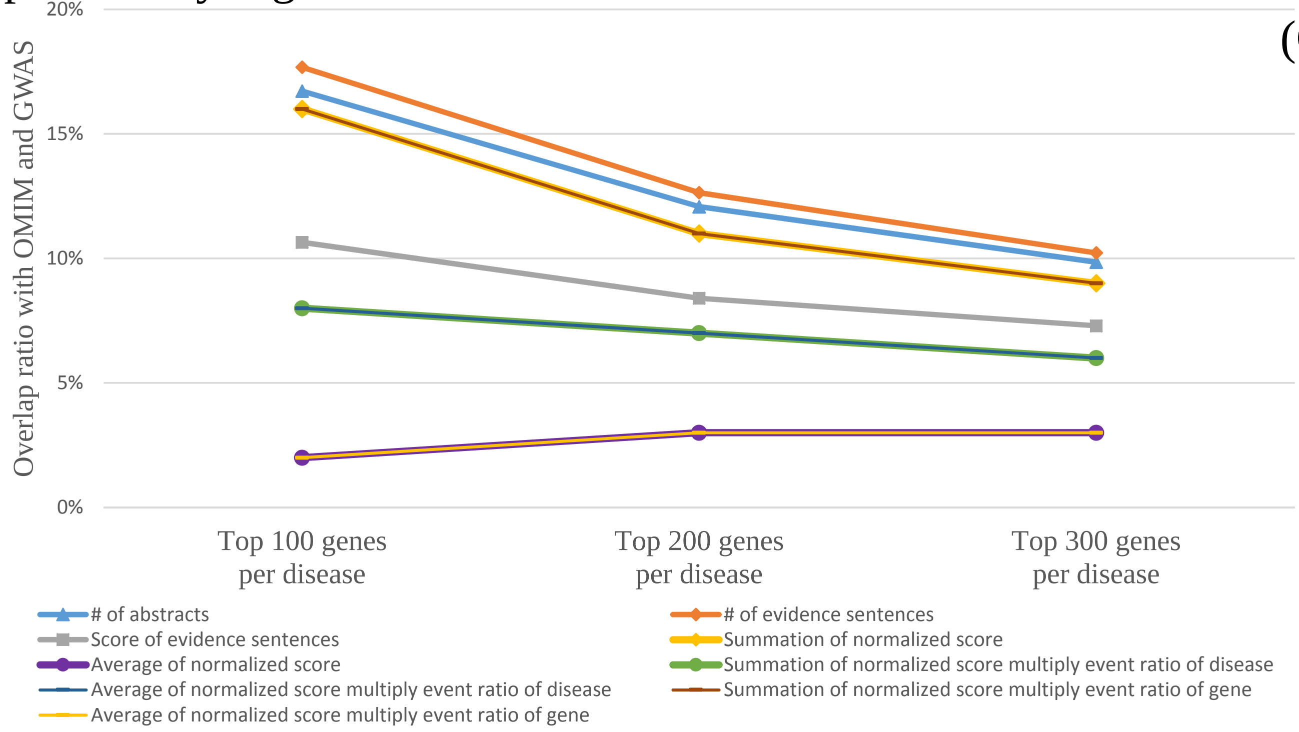

(C)

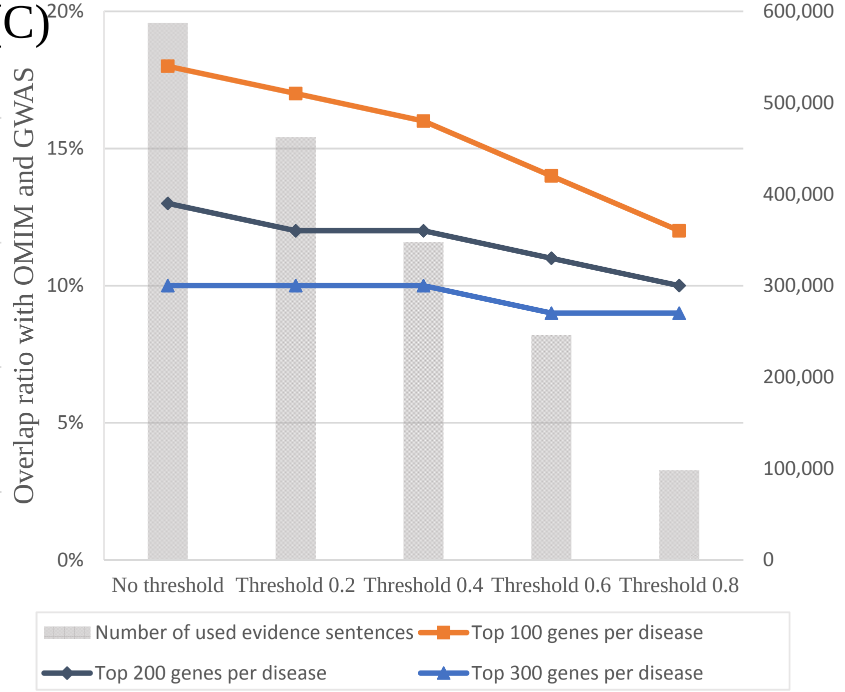

(B)

	# of abstracts	# of evidence sentences	Score of evidence sentences	Summation of normalized score	Average of normalized score	Multiply event ratio of disease		Multiply event ratio of gene	
						Summation of normalized score	Average of normalized score	Summation of normalized score	Average of normalized score
Top 100 genes	17%	18%	11%	16%	2%	8%	8%	16%	2%
Top 200 genes	12%	13%	8%	11%	3%	7%	7%	11%	3%
Top 300 genes	10%	10%	7%	9%	3%	6%	6%	9%	3%

Supplementary Figure 2

(A)

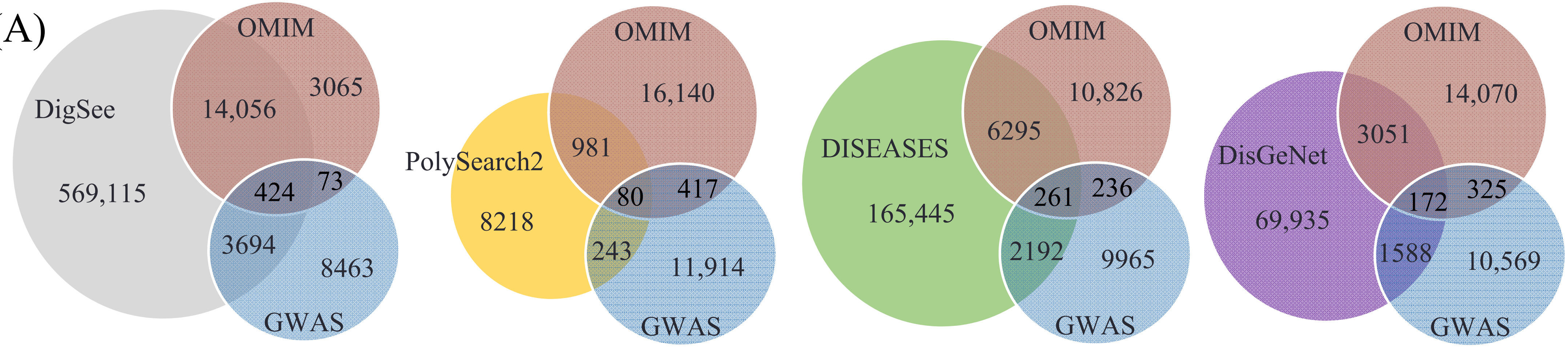

(B)

Distribution of coverage of disease-related genes

	PolySearch2		DISEASES		DisGeNet		DigSee	
	OMIM	GWAS	OMIM	GWAS	OMIM	GWAS	OMIM	GWAS
1.00	2	0	17	4	23	4	26	1
1.00~0.75	2	0	57	1	25	16	165	0
0.75~0.50	13	2	64	11	38	18	76	7
0.50~0.25	40	5	29	70	48	21	14	114
0.25~0.10	50	29	8	39	40	38	2	74
0.10~0.00	89	76	9	17	38	46	0	19
0	89	119	6	20	47	66	2	17
Not searched	4	3	95	70	26	23	0	0

(C)

Number of diseases having confidence overlap (p-value ≤ 0.05)

	OMIM	GWAS	OMIM + GWAS
PolySearch2	192	106	220
DISEASES	182	138	195
DisGeNet	210	139	231
DigSee	283	198	292

Supplementary Figure 3

(A) Separation coefficients calculation with top 100 genes

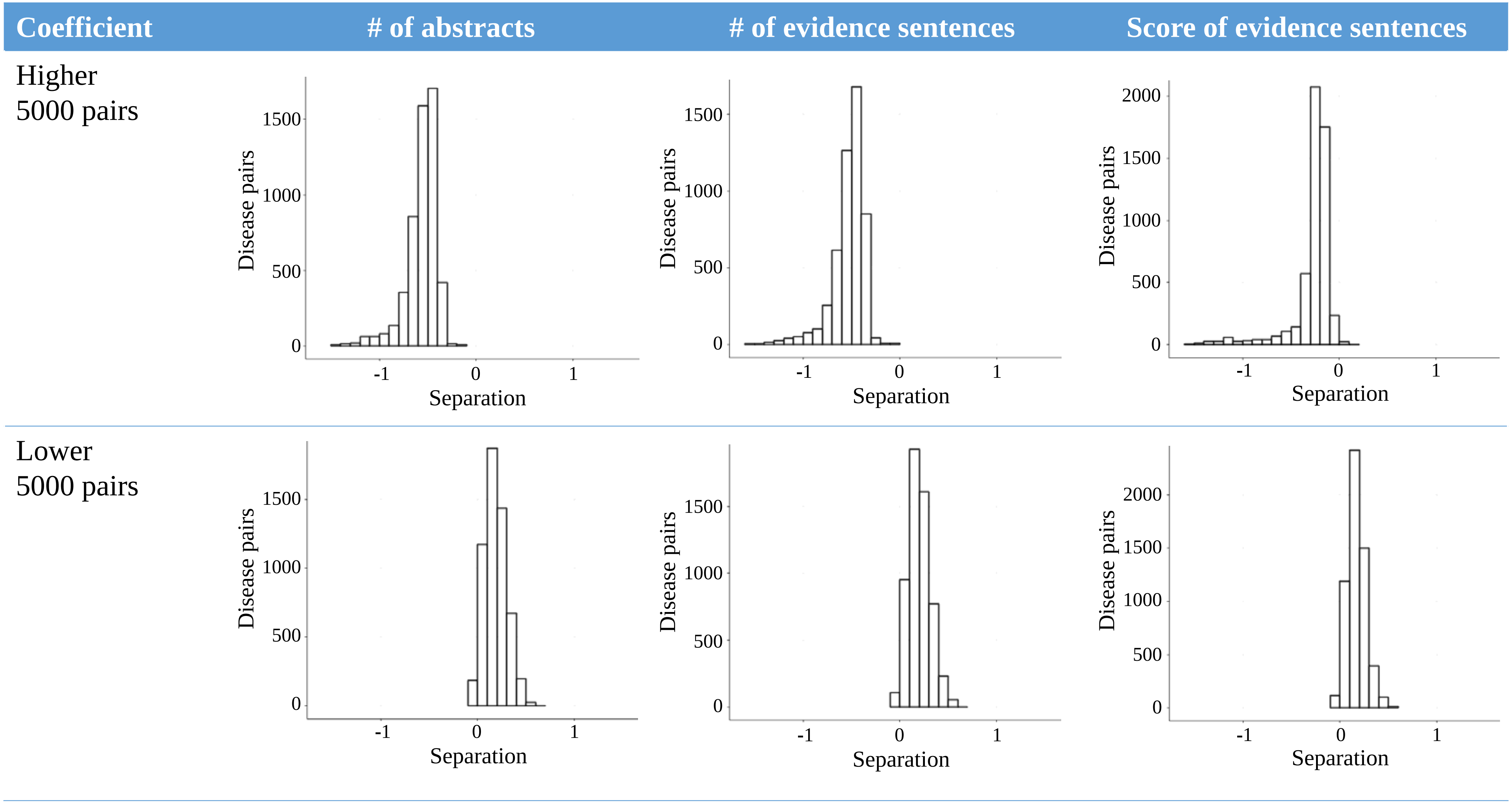

(B) Separation coefficients calculation (Ranked by evidence sentences)

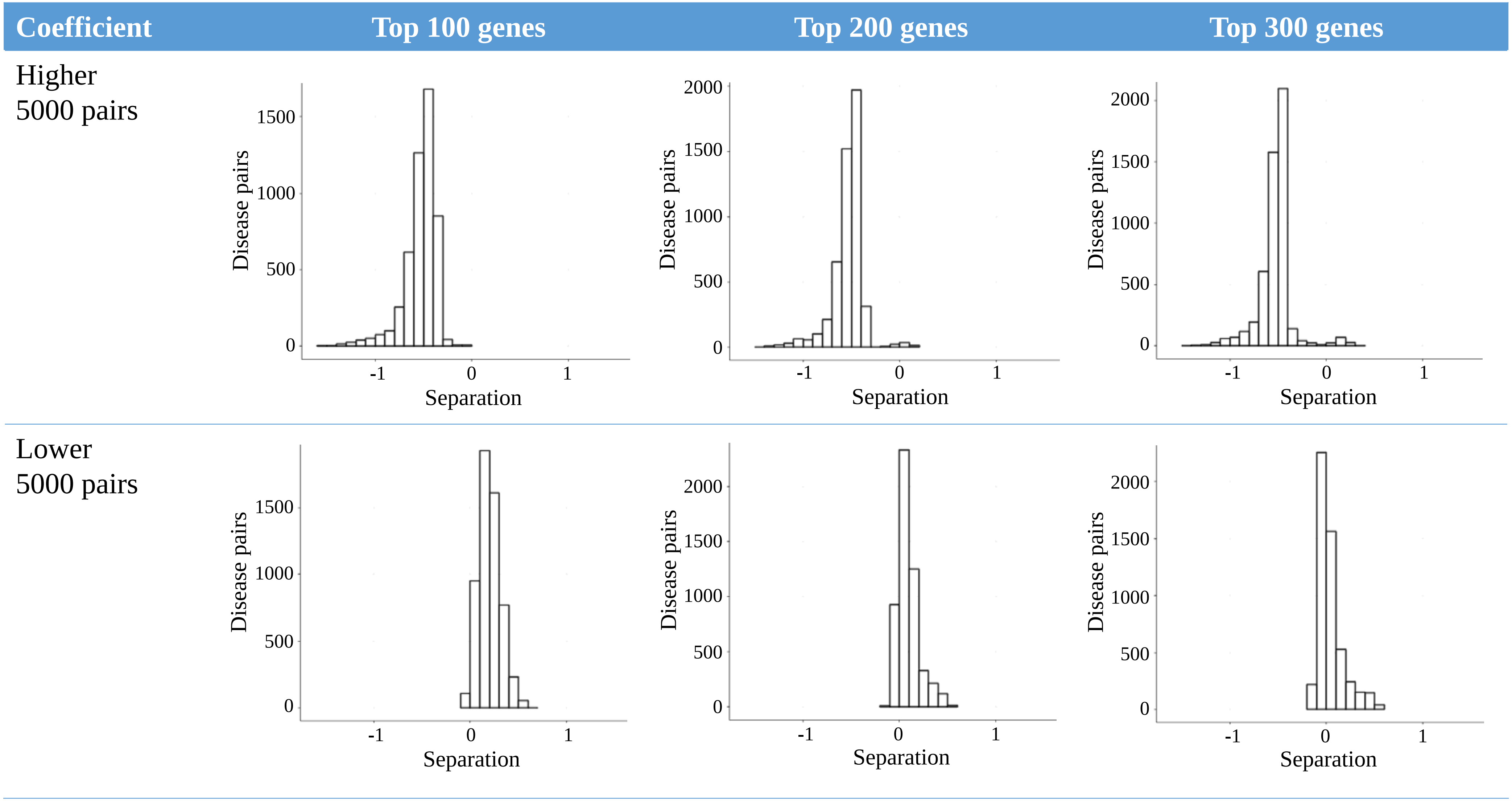

(C) GO term similarity: Biological process

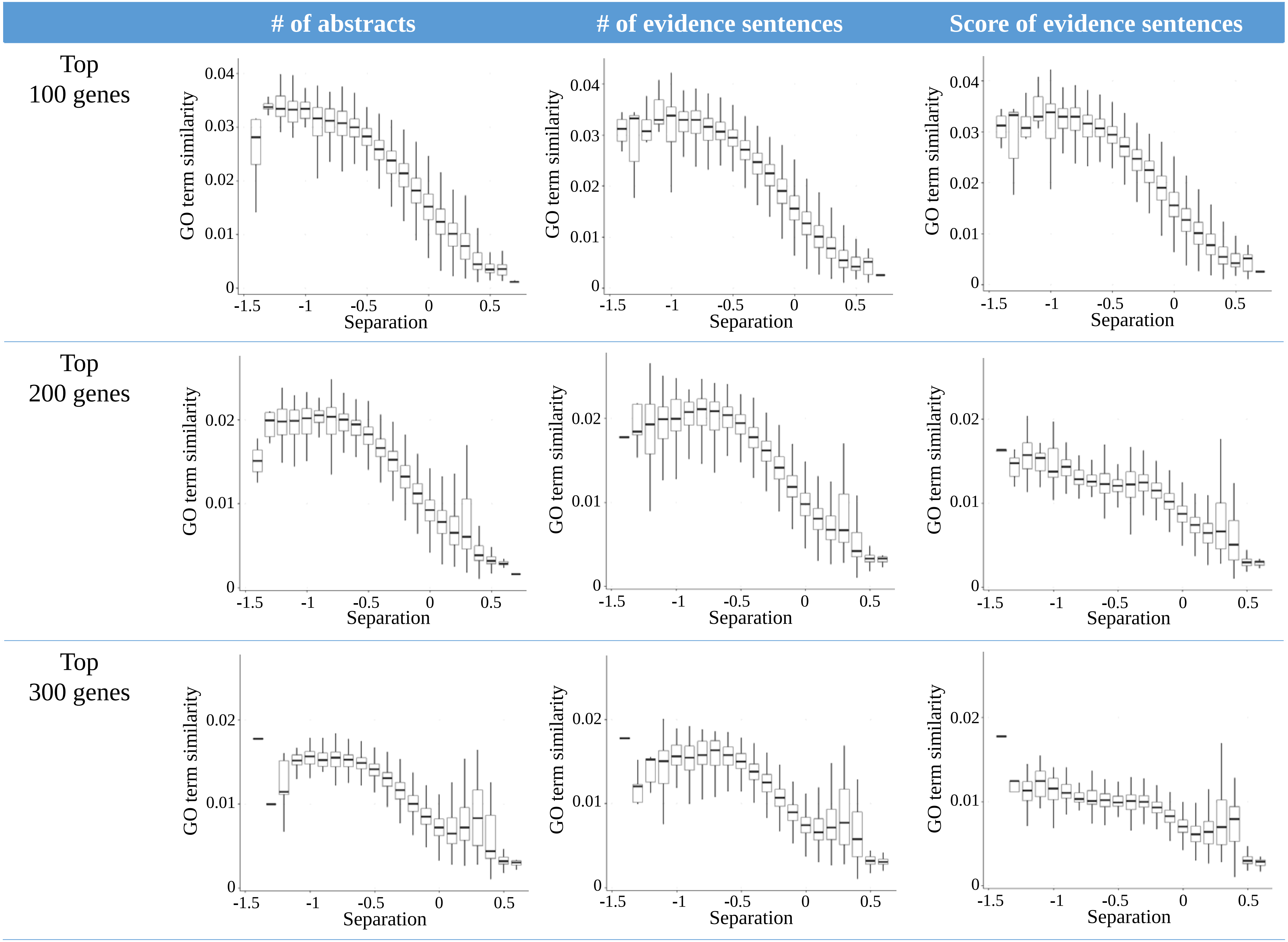

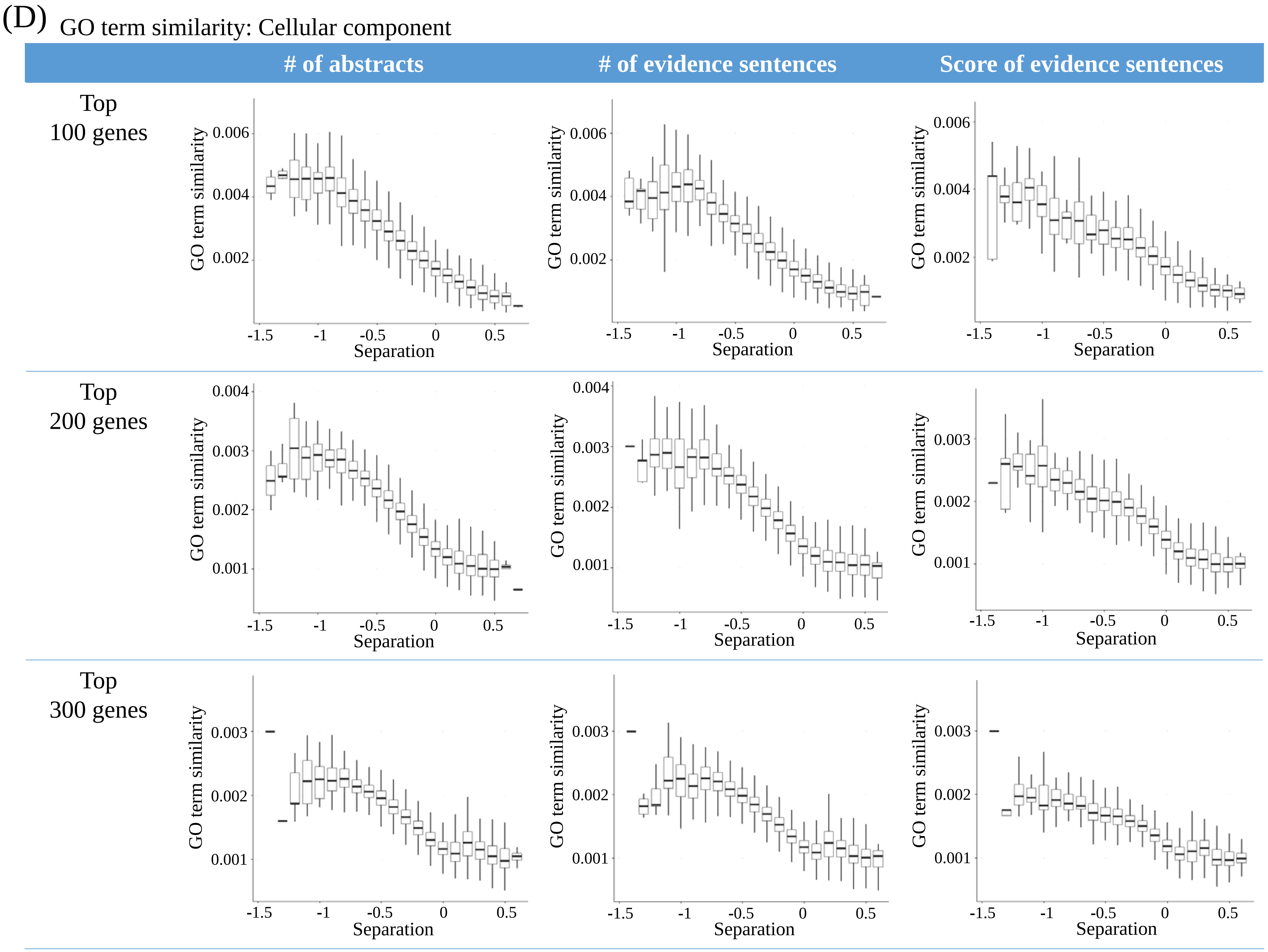
(D)
GO term similarity: Cellular component
# of abstracts
# of evidence sentences
Score of evidence sentences
Top
100 genes
Top
200 genes
Top
300 genes
GO term similarity
Separation
-1.5
-1
-0.5
0
0.5
0.006
0.004
0.002
0.003
0.001

(E) GO term similarity: Molecular function

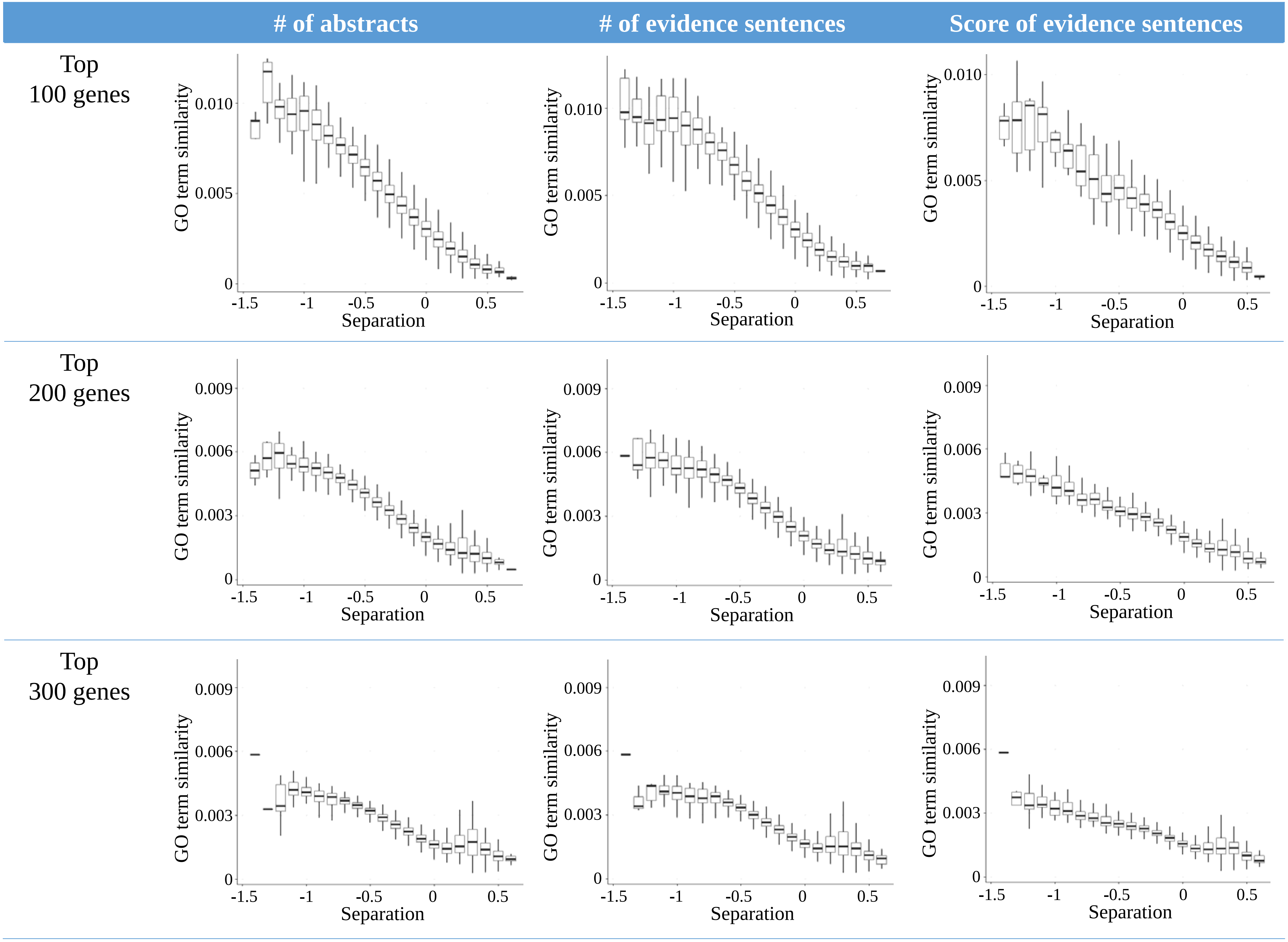

Supplement: Supporting Information [file srep40154-s1.pdf]
